# Cross-Directional Aggregation and Feature-Loss Optimization for Remote Sensing Semantic Segmentation

**DOI:** 10.3390/s26196196

**Published:** 2026-09-30

**Authors:** Chunbo Chang, Xinlin Xie, Shenran Guo, Yijie Zhang, Du Wang

**Affiliations:** 1 School of Electronic and Information Engineering, Taiyuan University of Science and Technology, Taiyuan 030024, China; ccb@tyust.edu.cn (C.C.); guoshenran@tyust.edu.cn (S.G.); s202215110524@tyust.edu.cn (Y.Z.); wangdu15@tyust.edu.cn (D.W.); 2 Shanxi Key Laboratory of Advanced Control and Industrial Intelligence, Taiyuan University of Science and Technology, Taiyuan 030024, China

**Keywords:** remote sensing, semantic segmentation, superpixels, neural networks, attention mechanism

## Abstract

**Highlights:**

**What are the main findings?**

- We propose a remote sensing semantic segmentation method with cross-directional aggregation and feature-loss optimization. The proposed method involves the state space model and superpixel edge optimization. The model includes three core modules: the two-stage cross-directional strip feature extraction module, hybrid attention-guided semantic injection module, and feature-loss dual optimization boosted superpixel edge module.
- We construct the Taiyuan Satellite Remote Sensing Dataset (TSRSD), which is a high-resolution benchmark covering diverse urban landscapes.

**What are the implications of the main findings?**

- Our proposed method integrates superpixels into a semantic segmentation deep learning framework to better segment complex scenes. The proposed method is suitable for various types and resolutions of high-resolution remote sensing images.
- Our proposed method demonstrates that the deep fusion of superpixels and semantic segmentation is the core approach to solving the problems of discontinuous and poorly fitted remote sensing segmentation boundaries, and it can accurately improve the boundary continuity of segmented objects.

**Abstract:**

The complexity of object categories in high-resolution remote sensing frequently gives rise to discontinuous boundaries in segmentation results. Furthermore, the inability to model global contextual morphological continuities along strip objects is a primary cause of segmentation fractures. Therefore, we propose a remote sensing semantic segmentation method with cross-directional aggregation and feature-loss optimization. First, we design a two-stage cross-directional strip feature extraction module (TCSFE), which is dedicated to modeling the global contextual information of strip objects. Specifically, our proposed module employs cross-directional attention interactions to capture global dependencies along both the horizontal and vertical directions. Second, we construct a hybrid attention-guided semantic injection module (HAGSI), which employs a dual-branch collaborative mechanism that integrates hybrid attention to enhance long-range feature modeling. Finally, we propose a feature-loss dual optimization boosted superpixel edge module (FDOSE). It employs feature fusion optimization and unified superpixel loss optimization to jointly optimize the final segmentation result. Experimental results on the Potsdam and Vaihingen datasets and the self-built TSRSD (Taiyuan Satellite Remote Sensing Dataset) demonstrate that our proposed method achieves state-of-the-art (SOTA) performance on the mIoU and Recall metrics, with mIoU scores of 79.93%, 80.25%, and 64.89% on Potsdam, Vaihingen, and TSRSD, respectively, and Recall scores of 87.70%, 88.94%, and 73.25%, respectively. Moreover, it exhibits an advantage in boundary continuity.

## 1. Introduction

Semantic segmentation yields a deep understanding of image content via per-pixel label assignment, a mechanism that facilitates fine-grained recognition at the pixel level [1]. Remote sensing semantic segmentation aims to automatically identify and label different land cover categories within images, providing precise data support for environmental monitoring, urban planning, and agricultural management [2]. However, high-resolution remote sensing images often present complex background and foreground information, as well as significant scale variations and intricate feature categories [3]. Therefore, the spatial heterogeneity and class imbalance inherent in remote sensing images contribute significantly to the challenge of achieving accurate segmentation [4]. Notably, urban scenes are extensively covered in remote sensing images, often containing numerous strip objects such as roads, waterways, and buildings.

Accurate object boundary delineation plays a paramount role in remote sensing analysis, particularly for linear geospatial features such as roads and rivers, which carry crucial geospatial information. However, existing semantic segmentation methods frequently encounter difficulties in preserving fine-grained object boundaries, notably in the case of strip objects. This pervasive limitation not only results in discontinuous segmentation outputs but also gives rise to severe segmentation fractures. To address the above issue, convolutional neural networks (CNNs) have been widely adopted to mitigate the above limitations by leveraging multi-scale feature extraction and boundary-aware modules. For instance, Wang et al. [5] designed a boundary-guided multi-scale feature fusion module to enhance the preservation of boundary information in strip features; He et al. [6] integrated a hollow space pyramid pooling module to extract multi-scale road features and enhance segmentation performance; Lu et al. [7] designed parallel convolutional kernels of varying sizes, enabling more efficient extraction of road information by integrating multi-scale features of the road; Wang et al. [8] proposed a dual-branch attention-guided and bidirectional feature enhancement network that utilizes dual-branch high- and low-frequency attention to extract edge details and global context, respectively, thereby enhancing the ability to distinguish the target from complex backgrounds. However, the inherently limited receptive field of CNNs prevents the effective capture of long-distance spatial dependencies between geographic spatial elements. Subsequently, Liu et al. [9] and Dosovitskiy et al. [10] proposed Swin Transformer and Vision Transformer to capture long-range interactions more efficiently. However, current methods remain confined to localized feature extraction modes, with insufficient capacity to model comprehensive feature correlations, which results in the loss of fine structural information.

The loss of spatial and structural information during feature extraction has necessitated the development of various auxiliary feature extraction mechanisms to augment feature representation. For example, Li et al. [11] proposed an attention-focused feature enhancement network, which gradually expands the receptive field via channel compression and self-residual scale expansion, strengthening its ability to extract multi-scale features; Wei et al. [12] proposed a feature-enhanced synthetic aperture radar (SAR) image segmentation method to address noise interference and boundary blurring in images; Shou et al. [13] proposed a graph information bottleneck module for remote sensing segmentation to enhance the feature representation of irregular geographic objects; Feng et al. [14] proposed a dynamic feature fusion module based on region query, which performed dynamic feature fusion based on region queries to enhance feature representation. However, most of them still use CNN and Transformer architectures, which are inefficient for modeling global contextual dependencies due to their quadratic computational complexity. Notably, Gu et al. [15] proposed the Mamba model based on state space models (SSMs), which effectively captures fine-grained local features while achieving linear computational complexity and efficiently modeling global semantic information. However, existing methods have primarily focused on Mamba’s ability to extract global features at the expense of its capacity for capturing local fine-grained details.

Existing multi-scale fusion designs historically emphasize local-to-global aggregation at the cost of the explicit global context needed for long-range continuity. As a result, the model produces disjointed boundaries, unable to infer that spatially separated segments belong to the same object class. To mitigate the above issue, several advanced frameworks have been proposed to incorporate superpixel segmentation into semantic segmentation. For example, Wang et al. [16] proposed the superpixels with a minimum spanning tree (SMST) network architecture, which performed initial oversegmentation of remote sensing images to generate superpixels, and then segmented the remote sensing images through clustering; Ren et al. [17] designed the region-sensitive adaptive superpixel generation and hybrid edge correction (RSASGEC) network, which enhanced image segmentation by constraining complex image boundaries through complex region-sensitive dynamic superpixel initialization; Li et al. [18] proposed a superpixel-based evolutionary multi-task feature selection method that improves classification accuracy via a multi-task optimization mechanism. However, superpixel-based methods typically limit the use of superpixels to preprocessing and often lead to ambiguity between superpixel edges and actual boundaries.

Motivated by the above discussion, we propose a remote sensing semantic segmentation method with cross-directional aggregation and feature-loss optimization. Inspired by the capability of state-space models to capture long-range spatial dependencies and enhance fine-grained detail representation, we design a hybrid attention-guided semantic injection module, which is built upon a state-space model, serving as an auxiliary branch to the main feature extraction network. Furthermore, we design a feature extraction module specifically for strip objects within the main feature extraction network. Finally, we incorporate superpixels into the network, which leverages superpixel feature fusion and boundary guidance to optimize segmentation boundaries. Extensive experiments demonstrate that our proposed method achieves superior performance on multiple benchmark datasets.

Our main contributions are as follows:

(1) TCSFE Module: We design a two-stage cross-directional strip feature extraction module. Our proposed TCSFE module captures global contextual information along both horizontal and vertical axes and ensures boundary continuity of strip objects by employing dedicated directional convolutions.

(2) HAGSI Module: We construct a hybrid attention-guided semantic injection module. Our proposed HAGSI module captures long-range dependencies and preserves fine-grained spatial details while capturing high-level semantic features by employing a dual-branch collaborative mechanism and a parallel structure of hybrid attention.

(3) FDOSE Module: We propose a feature-loss dual optimization boosted superpixel edge module, which uses a dual optimization mechanism of feature fusion optimization and joint superpixel loss optimization to jointly optimize the segmentation results.

(4) TSRSD Dataset: The proposed TSRSD provides a high-resolution urban remote sensing benchmark with dense and fine-grained annotations. It presents challenges unique to developing urban regions, specifically high intra-class variance, high inter-class similarity, and complex shadows, while facilitating rigorous evaluation of semantic segmentation models in heterogeneous urban environments and highlighting their generalization capability.

## 2. Related Work

### 2.1. Semantic Segmentation of Remote Sensing Based on Superpixels

Superpixels group pixels into boundary-adherent regions, offering a more holistic data representation than individual pixels. In remote sensing, using superpixels as processing units for object-oriented semantic segmentation leverages such spatial coherence to preserve structural detail. Thus, superpixel segmentation serves as a key preprocessing step for effective region-level feature extraction and classification. For example, Zhang et al. [19] proposed a graph model that enhances classification performance by effectively modeling spatial dependencies at the superpixel level. Jia et al. [20] proposed a multi-scale superpixel kernel that achieved high-precision classification by generating superpixels at different scales. However, these approaches of partitioning followed by classification suffer from fragmentation issues. To address this problem, existing work has proposed integrating superpixel segmentation and classification tasks into a single framework. For example, Li et al. [21] designed a cascaded superpixel model that achieved mutual reinforcement between superpixel segmentation and classification tasks through alternating optimization. Tang et al. [22] proposed a learnable superpixel generation module that used a UNet to generate pixel-superpixel association maps and superpixel features. Wang et al. [16] employed the SLIC method for oversegmentation, producing superpixels noted for their boundary adherence and noise resistance. Yang et al. [23] employed the SLICm superpixel method to segment remote sensing images, which extracts superpixels with well-defined boundaries and statistically analyzes the proportion of internal semantic categories to optimize the initial segmentation results. However, superpixel-based approaches require multiple iterations and are computationally inefficient. Xu et al. [24] point out that traditional superpixel algorithms require multiple iterations of clustering, graph cuts, or boundary swapping to optimize the superpixel grid; this process is time-consuming, and the discrete operations involved prevent integration into the network’s backpropagation process. Even when a differentiable approach is used to integrate the superpixel loss into the network in an end-to-end manner, the computational overhead remains significantly higher than that of single-stage FCN-style integration. To improve the segmentation results, we combine feature fusion optimization and joint superpixel loss optimization in this paper to form a superpixel edge optimization method.

### 2.2. Semantic Segmentation of Remote Sensing Based on State Space Models

CNNs and ViTs have been reported to exhibit certain limitations in expanding their receptive fields. For example, when multiple receptive fields are used for multi-scale feature fusion, similar information may be redundantly represented across scales. Moreover, global context modeling with ViTs can incur substantial computational costs, particularly for high-resolution inputs or long token sequences. In recent years, a novel network architecture based on selective state space models (SSMs), known as Mamba [15], has pioneered a new path in the field of computer vision [25]. Notably, Mamba employs variable parameters to represent global dependencies and leverages hardware-optimized computation methods to enhance computational performance. The Mamba architecture leverages the high efficiency and global modeling capabilities of state-space models in processing sequential data. For example, Liang et al. [26] proposed a bidirectional Mamba module, which optimizes the fusion of local details and global features by capturing long-distance dependencies, ensuring the effective preservation of key information. Li et al. [27] introduced a state-space model for semantic segmentation of remote sensing and proposed a semantic segmentation framework for high-resolution remote sensing images as an encoder. Zhu et al. [28] proposed UNet-Mamba, which used a Mamba decoder to handle complex high-resolution information and a local supervision module to enhance detailed perception. Sheng et al. [29] proposed a cross-attention spectral-spatial Mamba module, which effectively extracts global spectral-spatial features using the linear-complexity global modeling capabilities of Mamba. Fu et al. [30] proposed a multi-scale graph state-space network for satellite telemetry anomaly detection, enhancing the separability between normal and anomalous patterns as well as the capability for temporal localization of anomaly intervals. However, existing Mamba-based segmentation methods lack explicit mechanisms to suppress noise-correlated features [31], which can undermine their robustness in high-noise or complex-feature scenarios. To address this, our module employs similarity-based error signals to guide the optimization, and we demonstrate that it maintains accurate segmentation under high-noise conditions. Furthermore, complex data frequently leads to inaccurate and error-prone segmentation edges. Nevertheless, our proposed method employs dual-branch collaborative feature enhancement to improve modeling capabilities and utilizes hybrid attention to capture fine-grained features, thus addressing the challenge of fine-grained feature modeling.

## 3. Method

### 3.1. Overall Architecture

**Figure 1:**
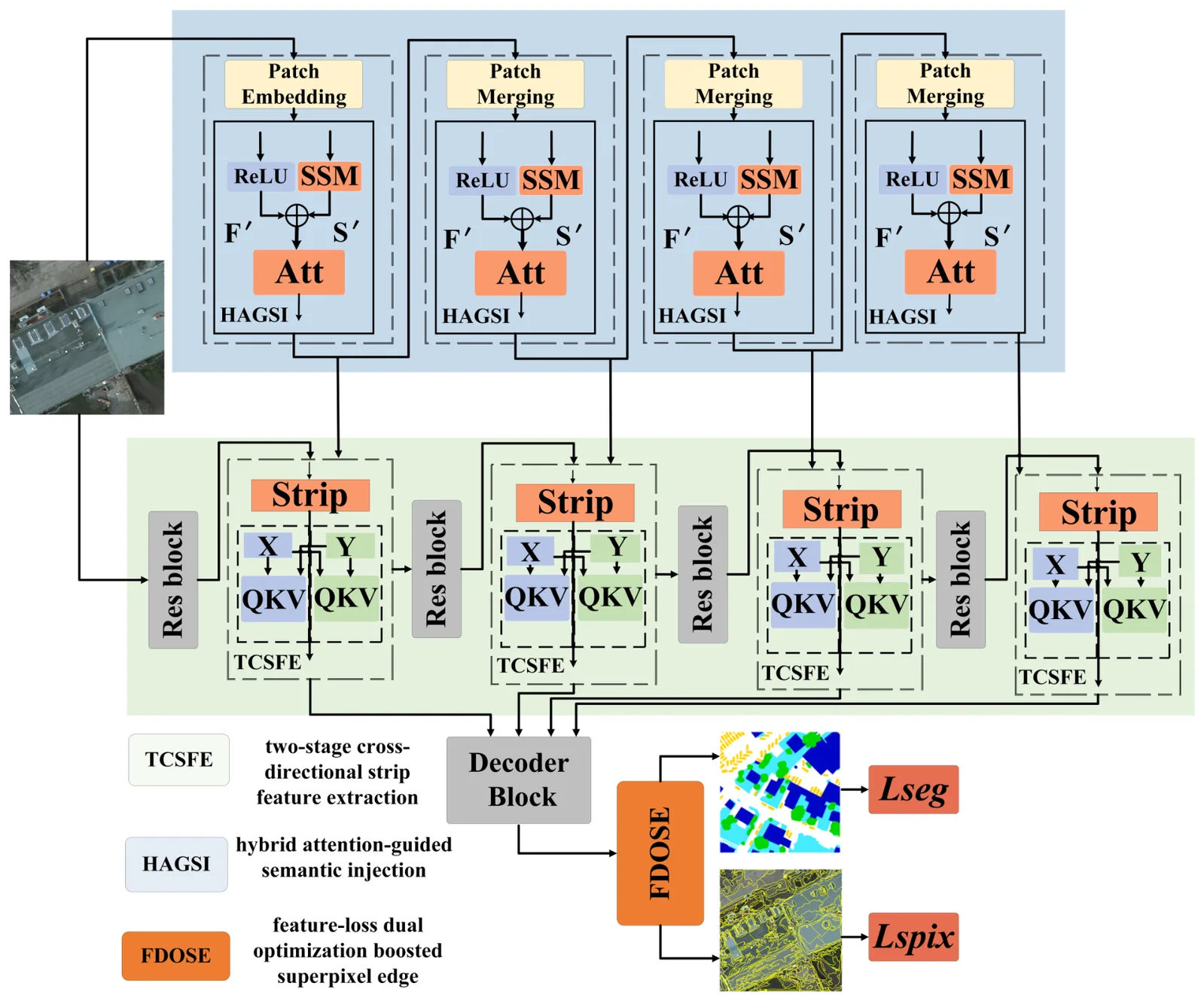
Framework of remote sensing semantic segmentation method with cross-directional aggregation and feature-loss optimization.

To address the issues of fractured segmentation of strip objects, as well as pervasive boundary discontinuity, we propose a remote sensing semantic segmentation method with cross-directional aggregation and feature-loss optimization. The overall network architecture of our proposed method is described in Figure 1.

The overall architecture of the method adopts an encoder–decoder structure, in which the backbone network of the encoder part uses ResNet-18 to extract multi-scale features of remote sensing as multi-level feature representations. In the decoding stage, the decoder fuses and parses the multi-scale information extracted by the encoder. Furthermore, the decoder effectively integrates features from different levels through a cross-layer feature alignment mechanism, which ensures full fusion of information between low-level and high-level features and improves the precision and accuracy of the segmentation results. The proposed method consists of three main components: a two-stage cross-directional strip feature extraction module (TCSFE), a hybrid attention-guided semantic injection module (HAGSI), and a feature-loss dual optimization boosted superpixel edge module (FDOSE).

### 3.2. Two-Stage Cross-Directional Strip Feature Extraction Module

**Figure 2:**
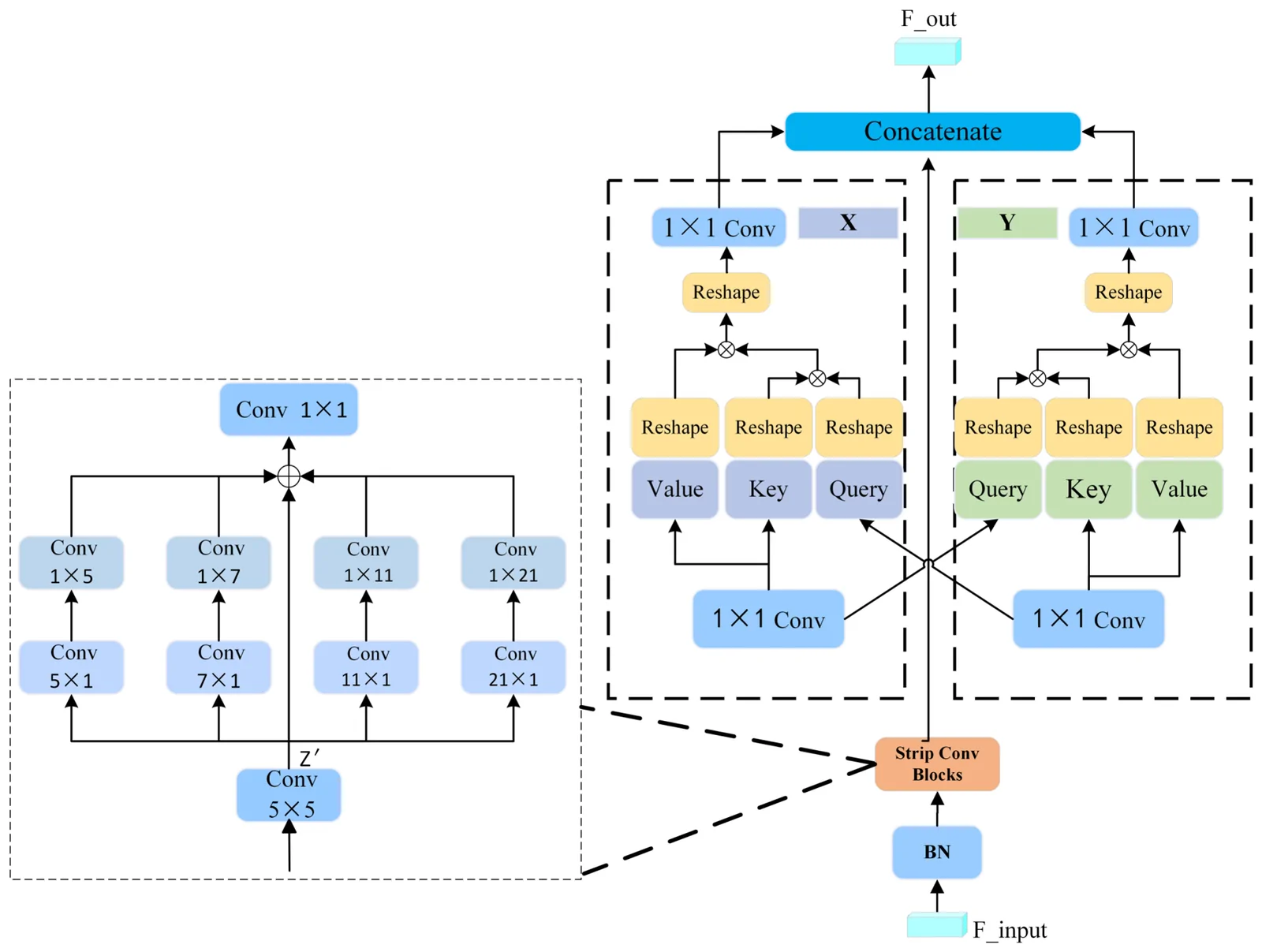
Structure diagram of TCSFE.

Downsampling in feature extraction networks often loses shallow spatial details, which hinders the capture of continuous strip structures. Existing symmetric convolution kernels, effective for global features, inadequately represent strip objects. Consequently, this inadequacy produces fractured or blurred segmentation depending on object orientation. Therefore, to address the issues of lost low-level semantic features and fractured segmentation of strip objects, we propose a two-stage cross-directional strip feature extraction module (TCSFE). The proposed TCSFE enhances shallow information representation and improves the segmentation accuracy of strip objects. The specific structure of the proposed module is shown in Figure 2.

The proposed TCSFE can deeply mine the edge information and local features of objects, extract and fuse features of low-level spatial details in different directions. Thus, the proposed TCSFE captures the morphological features of strip objects more accurately, effectively alleviating the problems of discontinuous segmentation and unstable boundaries in existing segmentation methods on strip objects. Our proposed TCSFE constructs a two-stage feature extraction mechanism featuring multi-scale, multi-directional operations and cross-directional attention fusion. The batch normalization process of the input features is shown in Equation (1): first, the input feature $F_{-}input$ is normalized to obtain feature $Z^{\prime }$; then, feature extraction is performed via a strip convolution block, aiming to enhance the model’s semantic representation capability for slender, strip objects. After obtaining $Z^{\prime }$, four parallel strip convolution branches with kernel scales 5, 7, 11, 21 are applied. Each branch first performs a k × 1 convolution and then a 1 × k convolution, so that vertical and horizontal strip responses at the same scale are jointly encoded. The outputs of the four branches are fused by element-wise summation to aggregate multi-scale strip features, and a 1 × 1 convolution is then used to recalibrate the fused channels and generate $F_{-}strip$. The feature fusion process of the strip convolutional block is defined in Equation (2), which comprises convolutional kernels of different scales and orientations, including 5 × 1 and 1 × 5, 7 × 1 and 1 × 7, 11 × 1 and 1 × 11, and 21 × 1 and 1 × 21. The configuration captures elongated features of different scales in both horizontal and vertical directions and thus avoids the limitations of a single convolutional kernel. This design enhances the representation ability of local information and ultimately generates an optimized feature map $F_{-}strip$. The formula is expressed as follows

(1) $$Z^{\prime }=Conv5\times 5\left(BN\left(F\_input\right)\right)$$

(2) $$F\_strip={\mathrm{Conv}}_{1\times 1}\left(\sum_{k\in \{5,7,11,21\}}{\mathrm{Conv}}_{1\times k}\left({\mathrm{Conv}}_{k\times 1}\left(Z^{\prime }\right)\right)\right)$$

where $F_{-}input$ represents the input feature, BN denotes the batch normalization operation, and $Z^{\prime }$ signifies the feature obtained after batch normalization; $F_{-}strip$ represents the feature output optimized by the strip convolutional blocks.

To model global dependencies and overcome single-direction attention limitations, we introduce a cross-directional interactive attention mechanism (X-axis and Y-axis) into the strip feature extraction module. The mechanism models spatial dependencies in different directions, integrates scattered local strip features, and fuses the optimized feature map $F_{-}strip$ from the strip convolution block. Our proposed cross-directional interactive attention mechanism can model global information across different directions, which enhances the ability to distinguish objects in segmentation tasks. Specifically, 1 × 1 convolutional kernels are used to reduce dimensionality and thus produce three matrices: query, key, and value. These matrices are then reshaped using tensor transformations to obtain attention scores. During the attention calculation process, cross-directional interaction is performed, such that the query values from the X-axis (horizontal) are input into the Y-axis (vertical) calculation, and the query values from the Y-axis (vertical) are input into the X-axis (horizontal) calculation, which results in interactive attention features $Attn_{-}X\left(Q,K,V\right)$ and $Attn_{-}Y\left(Q,K,V\right)$.

Finally, the two features are channel-adjusted through a 1 × 1 convolution and fused through concatenation, thus producing the final output feature map $F_{-}out$. The formula is expressed as follows

(3) X,Y=F_strip

(4) $$QKV\_X:\left\{\begin{array}{c} Q=W^{Q}Y\\ K=W^{K}X\\ V=W^{V}X \end{array}\right.$$

(5) $$QKV\_Y:\left\{\begin{array}{c} Q=W^{Q}X\\ K=W^{K}Y\\ V=W^{V}Y \end{array}\right.$$

(6) $$\left\{\begin{array}{cc} Attn\_X(Q,K,V) & =\mathrm{Reshape}\left(\mathrm{Softmax}\left(\frac{QK^{⊤}}{\sqrt{d_{k}}}\right)V\right)\\ Attn\_Y(Q,K,V) & =\mathrm{Reshape}\left(\mathrm{Softmax}\left(\frac{QK^{⊤}}{\sqrt{d_{k}}}\right)V\right) \end{array}\right.$$

(7) $$F\_out=\mathrm{Concat}\left(Attn\_X(Q,K,V),Attn\_Y(Q,K,V)\right)$$

where, QKV_X represents cross-directional projection operation to generate query, key and value for horizontal-axis attention; QKV_Y represents cross-directional projection operation to generate query, key and value for vertical-axis attention; $d_{k}$ represents feature dimension of projected query and key; Attn_X(Q,K,V) represents the attention output feature in the X-axis direction (horizontal); Attn_Y(Q,K,V) is the attention output feature in the Y-axis direction (vertical). The Query values are determined by cross-referencing the results of 1 × 1 convolutions. The design captures detailed information and enhances multi-scale perception through more efficient cross-directional feature utilization. The cross-directional information interaction effectively captures spatial dependencies in different directions. This improves segmentation accuracy for strip objects and achieves more continuous and boundary-aligned results.

### 3.3. Hybrid Attention-Guided Semantic Injection Module

**Figure 3:**
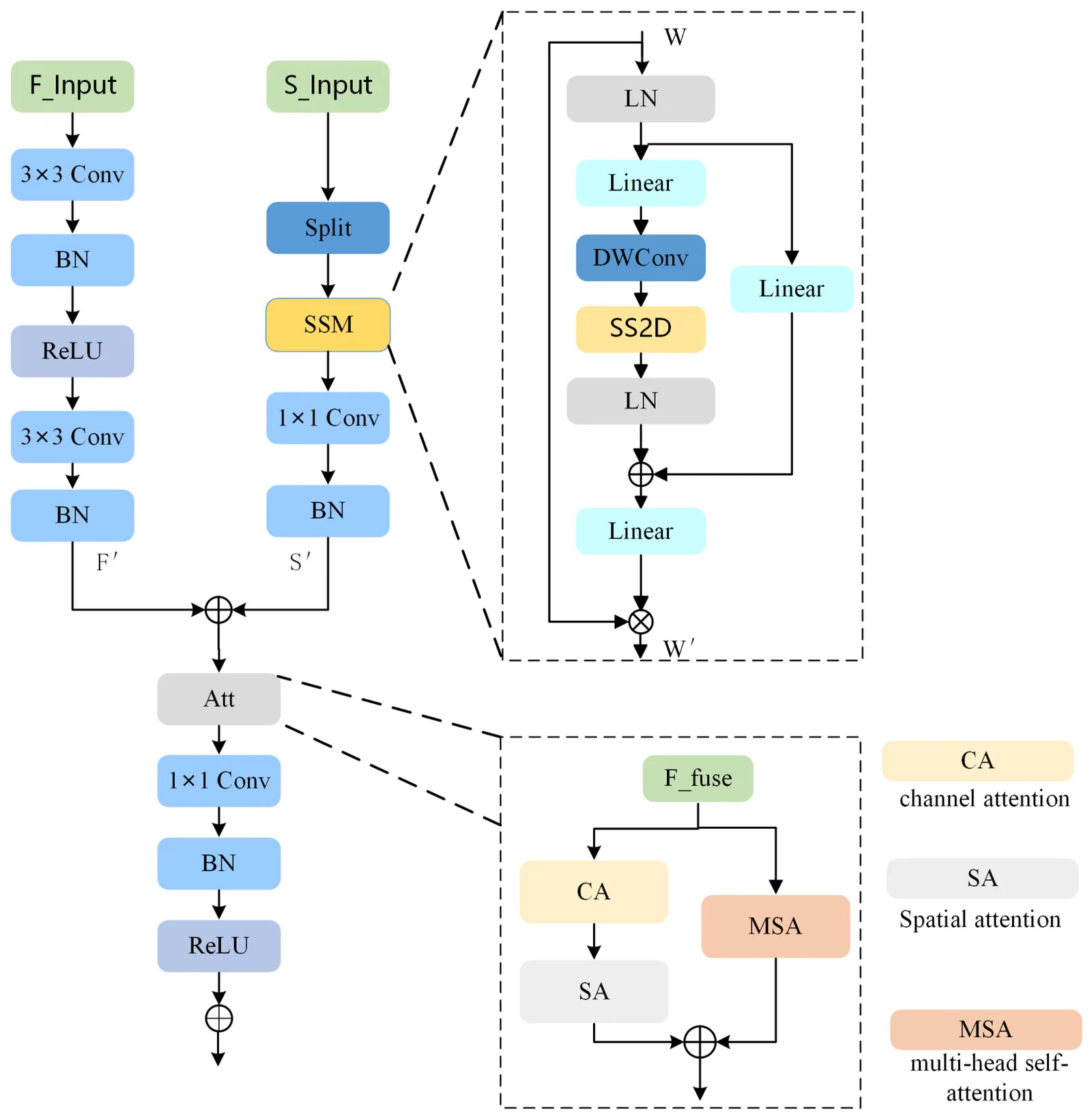
Structure diagram of HAGSI.

The inherent characteristics of remote sensing images limit the feature extraction capability of existing semantic segmentation networks. Different types of ground objects often exhibit high similarity in spectral characteristics and spatial distribution, which can easily cause class confusion in areas with blurry boundaries or similar texture features. To address the aforementioned issues, we propose a hybrid attention-guided semantic injection module (HAGSI). Our proposed module employs a dual-branch, collaborative multi-attention fusion enhancement architecture design. The proposed module enhances the spatial representation ability of long-range features through deep feature modeling and reduces interference from redundant information. Our proposed module effectively compresses feature representations and preserves key information, which improves the efficiency and accuracy of segmentation tasks. Furthermore, the proposed module optimizes semantic information representation in remote sensing against complex background interference. The structure of the proposed module is shown in Figure 3.

The proposed module takes two core inputs: (1) the initial features S_Input used for state-space assisted enhancement computation in each training batch; (2) the base feature map F_Input obtained through feature extraction by the backbone network. In the main feature map processing branch, a 3 × 3 convolutional kernel is first used to extract local features, followed by batch normalization and a ReLU activation function to enhance the model’s training stability and nonlinear expression capabilities.

Our proposed module contains an SSM branch, which takes the patch-embedded feature map S_Input and processes it using a state space model (SSM). The branch consists of two paths: one path processes the features through a normalization layer, followed by further optimization using deep convolution and a 2D Selective Scan (SS2D). The SS2D module works by assigning weights based on spatial feature importance. First, a convolutional operation compresses the feature map output from the deep convolution into a single-channel attention map. Then, an activation function generates importance weights for each spatial pixel. Subsequently, the weight map is multiplied element-wise with the original feature map, preserving high-weighted effective spatial features and suppressing low-weighted redundant background features. The designed module ultimately achieves spatial-dimension feature selection and optimization, which boosts the performance of subsequent model construction. The other path is processed only through a linear mapping layer. The output feature maps from both paths are then fused via pixel-wise addition, which enhances the modeling of long-range dependencies and ensures the network’s ability to capture global information. The formula is expressed as follows

(8) F′=BN(Conv3×3(ReLU(BN(Conv3×3(F_Input)))))S′=BN(Conv1×1(SSM(Split(S_Input))))F_fuse=F′⊕S′

(9) $$W^{\prime }=\mathrm{Linear}\left(\mathrm{LN}\left(\mathrm{SS}2D\left(\mathrm{DWConv}\left(\mathrm{Linear}\left(\mathrm{LN}\left(W\right)\right)\right)\right)\right)+\mathrm{Linear}\left(\mathrm{LN}\left(W\right)\right)\right)⊗W$$

where S_Input is the output of the Patch Embedding, serving as the input feature for the SSM auxiliary branch; F_Input is the feature map extracted from the backbone network; Conv3×3 represents a 3 × 3 convolution operation; Conv1×1 represents a 1 × 1 convolution operation; BN represents the normalization operation; ReLU is the activation function; Split represents the partitioning operation. SSM is the state space selection module; $S^{\prime }$ represents the output feature map of the auxiliary branch; $F^{\prime }$ is the main network output feature map; ⊕ represents pixel-by-pixel addition; F_fuse represents the result of adding the pixels together. Linear is a linear mapping; LN represents layer normalization.

The outputs from the main and SSM branch are fused via pixel-wise addition, which enhances the complementarity of multi-source information. Feature enhancement is performed through an attention module (Att), which combines channel attention (CA), spatial attention (SA), and multi-head self-attention (MSA) in parallel to enhance global feature modeling capabilities. Specifically, CA focuses on task-relevant channels by compressing spatial features into channel vectors with global average pooling and then determining their importance through a fully connected layer. SA generates dual-channel feature maps through channel-wise max and average pooling, which are then fused by a convolutional layer into a single-channel spatial weight map that pinpoints key spatial regions of ground features. MSA splits the feature map into multiple attention maps and computes the relationships between different spatial locations to capture long-range information. The attention output feature formula is expressed as follows

(10) F_Att=SA(CA(F_fuse))⊕MSA(F_fuse)

where F_fuse represents the result after feature fusion, CA represents channel attention, SA represents spatial attention, MSA represents multi-head self-attention, and F_Att is the final output feature map.

By capturing and integrating contextual information over longer distances, the proposed module network can focus on key regions and parts, which yields attention output features. Then, the stability of the feature distribution is improved by using 1 × 1 convolution and normalization layers, as well as the ReLU activation function, thus generating a high-precision feature map output.

### 3.4. Feature-Loss Dual Optimization Boosted Superpixel Edge Module

**Figure 4:**
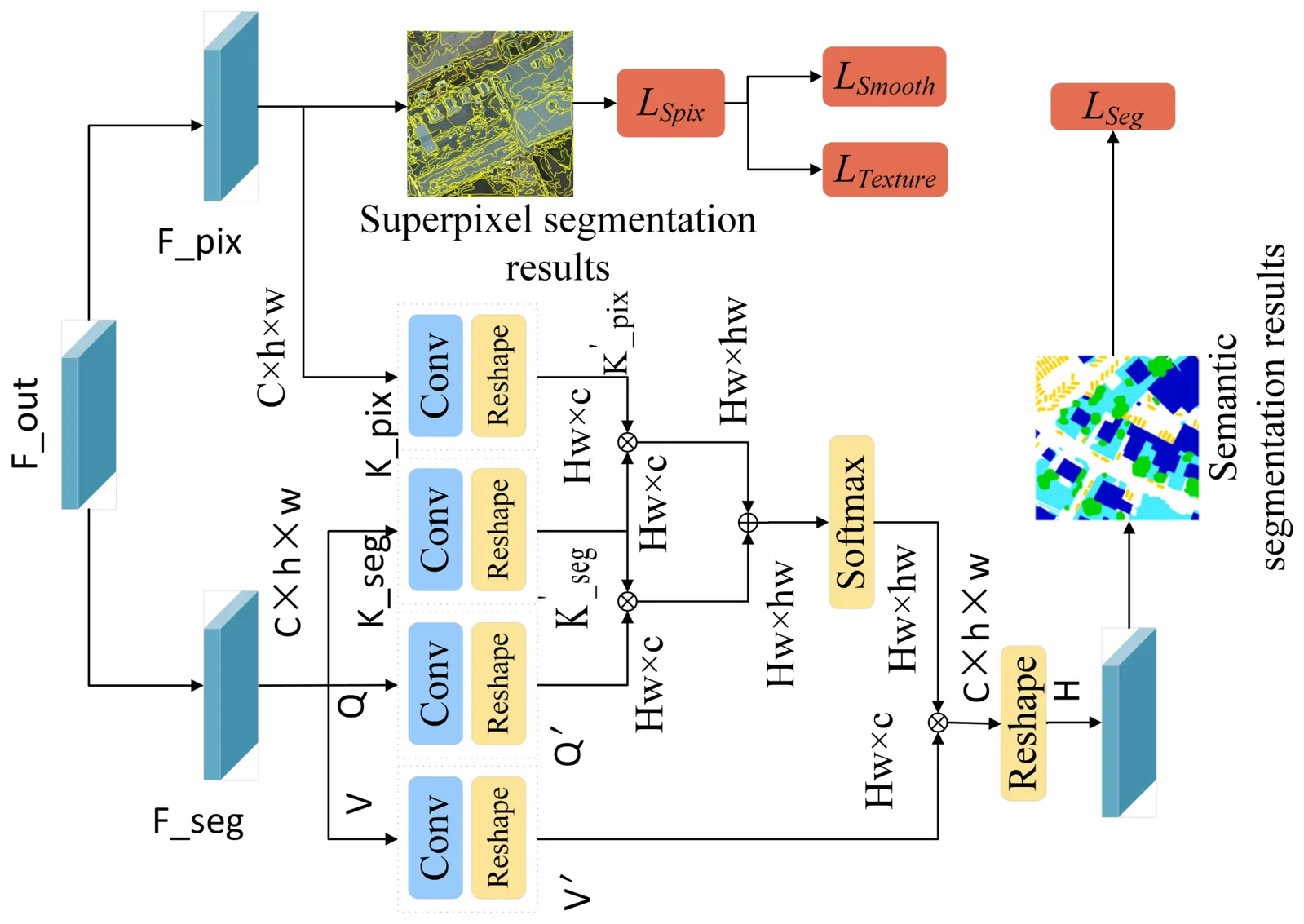
Structure diagram of FDOSE.

We propose a feature-loss dual optimization boosted superpixel edge module (FDOSE). The proposed module integrates superpixel and semantic segmentation features via a shared backbone to improve accuracy. Specifically, the module uses superpixel loss as a constraint, subsequently outputting both segmentation results simultaneously through a shared neural network. Moreover, the precise boundary information provided by superpixel segmentation further enhances the deep features of semantic segmentation. The proposed module structure is shown in Figure 4.

(1) Feature Fusion Optimization

Feature fusion optimization generates superpixel features from backbone features F_out for injection into the final semantic segmentation layer to enhance the output. First, the superpixel extraction feature is defined as F_pix, and the semantic segmentation result feature is defined as F_seg. The number of channels is adjusted by performing a 1 × 1 convolution operation on the two output features, respectively. The feature map after convolution is reshaped through a Reshape operation to facilitate matching with other features. Subsequently, a standard self-attention mechanism adjusts the feature map and computes attention weights on the adjusted feature map; then, the superpixel feature F_pix serves as the key, while the semantic segmentation feature F_seg serves as the query, key, and value. Subsequently, the attention weights of the key value of F_pix and the query and key values of F_seg are calculated and weighted averaged. A SoftMax activation obtains the attention weights at each boundary position. The focus of the network is on important areas like object boundaries while suppressing the influence of irrelevant parts.

Finally, using the attention weights obtained from SoftMax, the value of F_seg is weighted and averaged to output the weighted features. Fusing feature maps from superpixel and semantic segmentation enhances global image information modeling. The fusion of features effectively suppresses interference from unimportant regions in complex scenes and improves segmentation accuracy. The formula is expressed as follows

(11) V′,Q′,K_seg′,K_pix′=Reshape(Conv1×1(V,Q,K_seg,K_pix))H=Reshape(Softmax(K_seg′⊗K_pix′+K_seg′⊗Q′)⊗V′)

where Reshape is the reshaping operation.

(2) Superpixel Loss Optimization

The feature extraction process integrates semantic segmentation and superpixels into a joint-loss-optimized network, which resolves lost details. The proposed superpixel segmentation loss comprises boundary smoothing loss and texture loss. The semantic segmentation loss employs cross-entropy loss. The two losses form a joint loss function. Specifically, boundary smoothing loss ensures the smoothness and continuity of segmentation boundaries; texture loss optimizes detailed information and reduces errors caused by texture blurring during segmentation. Nevertheless, cross-entropy loss guarantees the accuracy of semantic segmentation results.

Joint Loss Function: To achieve globally optimal segmentation results for remote sensing, we integrate boundary smoothing loss, texture loss, and semantic segmentation cross-entropy loss to jointly constrain network training. The joint loss function is expressed as follows

(12) $$L_{\mathrm{Smooth}}=\frac{1}{\left|N\right|}\sum_{(i,j)\in N}w_{ij}\left(1-\sum_{k=1}^{K}Q_{ik}Q_{jk}\right)$$

(13) $$L_{\mathrm{Texture}}=\frac{1}{K}\sum_{k=1}^{K}\frac{\sum_{i}Q_{ik}{∥T_{i}-{\bar{T}}_{k}∥}_{2}^{2}}{\sum_{i}Q_{ik}}$$

(14) $$L_{\mathrm{Spix}}=L_{\mathrm{Smooth}}+L_{\mathrm{Texture}}$$

(15) $$L_{total}=L_{Ce}+\alpha L_{Spix}$$

where $L_{total}$ is the combined loss, $L_{Spix}$ represents the superpixel loss, $L_{Ce}$ represents the cross-entropy Loss, and α is the weighting coefficient. $w_{ij}$ represents the neighborhood similarity weight between pixel *i* and pixel *j*, ${\bar{T}}_{k}$ represents the average texture feature of the k-th superpixel, and $Q_{ik}$ represents the soft assignment probability of pixel *i* belonging to the k-th superpixel. The weighting coefficient α for the joint loss is set to 0.35. The weighting coefficient α controls the balance between the primary cross-entropy loss and the auxiliary superpixel loss. The cross-entropy term provides the main semantic supervision, while the superpixel term acts as a boundary and texture regularization. If α is too small, the superpixel constraint is weakened, and boundary discontinuity may remain. If α is too large, the auxiliary regularization may dominate training and cause over-smoothing or missing small objects. Therefore, α is empirically set to 0.35 to keep semantic supervision dominant while providing sufficient boundary regularization. It was fixed in all experiments.

Boundary optimization and smoothing region adjustments are performed on semantic segmentation boundaries with superpixel segmentation results. This enhances boundary coherence and reduces misclassifications caused by blurred object boundaries. Additionally, the joint loss function is used to optimize the feedforward network, which enhances the ability to represent details and makes object boundaries better delineated.

## 4. Experimental Results and Analysis

To validate the effectiveness of our proposed method, we conducted experiments on semantic segmentation of remote sensing images. First, we introduced the experimental environment, network configuration, datasets, and evaluation metrics. Second, ablation experiments and method performance comparisons were conducted for each module. Finally, we conducted comparative experiments and visualizations against SOTA methods.

### 4.1. Experimental Environment and Network Configuration

All experiments were conducted using the PyTorch deep learning framework on a 64-bit Ubuntu 22.04 operating system with a 32GB GeForce RTX 3090 GPU. Data processing was performed with PyTorch 1.7.1 and Python 3.9. The Adam optimizer was employed during model training with a fixed sampling interval of 16, a patch size of 5, an initial learning rate of $8\times 10^{-5}$, weight decay set to $1\times 10^{-4}$, and a poly learning rate adjustment strategy. The batch size was configured to 4, with 100 epochs. A ResNet-18 backbone network was utilized as the encoder to extract deep features. The weighting coefficient α for the joint loss is set to 0.35.

### 4.2. Datasets and Evaluation Metrics

Experiments were conducted on the ISPRS Potsdam dataset, the ISPRS Vaihingen dataset, and a self-built high-resolution satellite remote sensing dataset of the Taiyuan urban area in Shanxi Province (Taiyuan Satellite Remote Sensing Dataset, TSRSD).

(1) ISPRS Potsdam dataset: The Potsdam dataset comprises 38 high-resolution aerial images (ground sampling distance of 5 cm) measuring 6000 × 6000 pixels. This dataset is annotated with six land cover types: Impervious Surface, Building, Low Vegetation, Tree, Car, and Clutter. The original tiles were first divided into training, validation, and testing subsets at the tile level and then cropped into 512 × 512 patches using a sliding window, yielding an approximate patch-level ratio of 8:1:1 for the training, validation, and testing sets. Data augmentation techniques applied during model training included horizontal and vertical flipping, random scaling, and others.

(2) ISPRS Vaihingen dataset: The Vaihingen dataset comprises 33 high-resolution images with an average size of 2494 × 2064 pixels and a spatial resolution of 9 cm. It shares the same category definitions as the Potsdam dataset. The dataset was split into training, validation, and test sets at an 8:1:1 ratio, following the same split protocol as the Potsdam dataset.

(3) TSRSD dataset: High-Resolution Satellite Remote Sensing Dataset for Taiyuan Urban Areas, Shanxi Province. The source imagery consists of domestically produced satellite images covering six urban districts of Taiyuan City, Shanxi Province. The image dimensions reach 56,251 × 52,654 pixels with a spatial resolution of 1 m. The TSRSD dataset uses Gaofen-2 satellite imagery as its primary data source. From January 2022 to 5 June 2023, a total of 646 scenes of original satellite imagery covering the six urban districts of Taiyuan were received. Using remote sensing image processing platforms such as GXL2018 and EasySat, multi-source satellite images within the urban area were sequentially subjected to control point collection, block adjustment, orthorectification, image fusion, and band recombination. After meeting the accuracy requirements, a 3-band, 8-bit true-color remote sensing image dataset was generated. For orthophoto production from Gaofen-2 and other satellite images with a resolution better than 1 m, the mapping accuracy requirements for 1:10,000 topographic maps were used as the reference. The dataset was manually annotated at the pixel level into 9 categories: farmland, tree, grass, water, building, hardened surface, excavated area, road, and background. In the experiment, the dataset was divided into training, validation, and test sets at an approximately 8:1:1 ratio, containing 5285, 661, and 661 images, respectively. Original images were cropped to 512 × 512 pixels to ensure optimal training, validation, and testing outcomes.

To better evaluate model performance, we employed four widely used evaluation metrics in the superpixel segmentation domain for the segmentation results: Under-segmentation Error (UE), Boundary Recall (BR), Achievable Segmentation Accuracy (ASA), and Mean Distance to Edge (MDE) [32].

For semantic segmentation results, we evaluated performance using the mean F1 score (mF1), F1 overall accuracy (OA), intersection over union (IoU), mean intersection over union (mIoU), and Recall. The formula is as follows

(16) $$\left\{\begin{array}{c} F1=2\times \frac{Precision\times Recall}{Precision+Recall}\\ mF1=\frac{1}{N}\sum_{k=1}^{N}{F1}_{k} \end{array}\right.$$

(17) $$\left\{\begin{array}{c} IoU_{i}=\frac{TP_{i}}{TP_{i}+FP_{i}+FN_{i}},\;i=1,\ldots ,N\\ mIoU=\frac{1}{N}\sum_{i=1}^{N}IoU_{i}=\frac{1}{N}\sum_{i=1}^{N}\frac{TP_{i}}{TP_{i}+FP_{i}+FN_{i}} \end{array}\right.$$

(18) $$OA=\frac{TP+TN}{TP+FP+TN+FN}$$

(19) $$Recall=\frac{TP}{TP+FN}$$

where Precision measures how many of the predicted positives are true positives, $Precision=\frac{TP}{TP+FP}$, and TP, FP, TN, and FN represent True Positives, False Positives, True Negatives, and False Negatives respectively. *N* is the number of categories. k∈[1,…,K] denotes the object with index k, $F1_{k}$ is the F1 score for category k.

### 4.3. Ablation Experiments

To verify the effectiveness of our proposed module, we conducted module-wise ablation experiments on the ISPRS Potsdam dataset. We employed a ResNet-18 encoder coupled with a decoder that uses upsampling and convolution, which served as the base, and then progressively incorporated the proposed TCSFE and HAGSI. Additionally, our proposed FDOSE was validated in ablation comparison experiments. The results of the module-wise ablation experiments are shown in Table 1.

As shown in Table 1, the performance metrics after adding the TCSFE module are significantly better than the base model, which indicates that this module can focus on strip features in different directions and significantly improve the accuracy of feature extraction for strip objects. Furthermore, after successively adding the HAGSI module to the “Base + TCSFE” model, all metrics are further substantially improved, which demonstrates that the HAGSI module can deeply capture long-distance dependencies and thus enhances the overall modeling capability. The integration of all three proposed modules into the baseline, denoted as “Base + TCSFE + HAGSI + FDOSE”, yields the best overall performance across all evaluation metrics. The results fully validate the effectiveness of each module.

#### 4.3.1. The Impact of TCSFE Module on Network Performance

The proposed TCSFE module aims to enhance the segmentation accuracy of strip objects by combining edge information with spatial details to produce sharp boundaries and improve segmentation precision. To validate the effectiveness of the proposed module, we selected classic feature extraction modules for ablation experiments. The feature extraction modules include FRH [33], SFE [34], CAFEM [35], and DFEM [36]. The symbol “-” indicates no additional feature processing. Table 2 shows the experimental results of the proposed strip feature extraction module. For the comparison in Table 2, the baseline does not employ any feature extraction module. Different feature extraction modules are then inserted into this same baseline for a fair comparison.

Table 2 demonstrates that the core metrics of the proposed two-stage strip feature extraction module with cross-directional attention outperform the feature extraction module in the comparison method. Notably, the Recall metric reached 83.09%, representing a substantial 8.97 percentage point improvement over the baseline without any feature extraction module. This significant enhancement is primarily attributed to the cross-directional attention design of the proposed module. Specifically, the design enables focused attention on strip features oriented in different directions, improving the extraction of strip features. Consequently, the network’s ability to recognize and represent strip objects is enhanced.

#### 4.3.2. The Impact of HAGSI Module on Network Performance

The proposed HAGSI module models deep features to enhance key features and reduce errors by optimizing spatial expressiveness. To validate the proposed module’s effectiveness, we conducted ablation experiments comparing it against classical auxiliary enhancement modules. The auxiliary enhancement modules included Swin [9] architectures, DFM [37], and FIA [38]. Table 3 shows the experimental results, where “-” denotes no branch or module processing applied. For the comparison in Table 3, the baseline does not employ any auxiliary feature enhancement module. Different auxiliary feature enhancement modules are then inserted into this same baseline for a fair comparison.

As shown in Table 3, the proposed HAGSI module outperforms the comparison module. Through SSM’s global dependency capture, the module strengthens edge delineation by mitigating boundary weakening and reducing blurring and discontinuity. Simultaneously, the built-in attention mechanism within HAGSI significantly enhances the global feature modeling capability, precisely locates key spatial regions of ground features, and further bolsters the global integrity and robustness of feature representation.

#### 4.3.3. The Impact of FDOSE Module on Network Performance

The superpixel fusion module combines the advantages of superpixel segmentation to assist the semantic segmentation network in extracting refined object boundaries. By removing the superpixel optimization module, the results are compared with the complete network. The experimental results are shown in Table 4. “-” indicates that the FDOSE module is not added, which is “Base + TCSFE + HAGSI”. Then, the effectiveness is demonstrated by adding the FDOSE module, and it is further measured by the indicators UE, BR, and ASA.

As shown in Table 4, the proposed FDOSE module enhances semantic segmentation results and significantly improves performance indicators by fusing superpixel information with deep features. Specifically, mIoU increased from 77.81% to 79.92%, and UE decreased from 0.2014 to 0.1535. This improvement mainly comes from the superpixel boundary optimization module’s ability to segment images into superpixel blocks with similar textures, extract detailed edges, optimize boundary details and complex areas, and better align with real object boundaries. Thus, FDOSE maintains object continuity and integrity, enabling our model to perceive global features. The experiment fully verifies the effectiveness of the superpixel boundary optimization module in optimizing boundary details.

### 4.4. Method Performance Analysis and Comparison

We compared state-of-the-art methods such as UNetFormer [33], PSPNet [39], Deeplabv3+ [40], ABCNet [41], RS^3^Mamba [42], CCTNet [43], Swin Transformer [9], SegFormer [44], LGBSwin [45], and SwinFAN [46]. To better evaluate model performance, semantic segmentation results are assessed using overall accuracy (OA), mean intersection over union (mIoU), F1 and Recall.

#### 4.4.1. Quantitative Analysis

The comparative experimental results are shown in Table 5, Table 6 and Table 7. Our proposed method achieves optimal results in all metrics, especially in complex boundaries, where the proposed method has a significant advantage and can achieve better segmentation results in the future. For all reported metrics, including mIoU, mF1, Recall, and OA, the clutter/background class is excluded from the computation.

The experimental results in Table 5, Table 6 and Table 7 demonstrate that our proposed method achieved the best performance metrics across all three datasets, significantly outperforming the comparison methods. Particularly, the OA metric reaches 89.76% on the Potsdam dataset. The superiority stems primarily from the proposed FDOSE module, which refines superpixel block edges to precisely assign pixels belonging to complex boundaries. Additionally, the cross-directional attention feature extraction effectively captures the continuous features of strip objects, comprehensively enhancing segmentation accuracy and integrity.

For the single-category F1 metric, our proposed method demonstrated exceptional segmentation capabilities, as shown in Table 5, Table 6 and Table 7. On the Potsdam dataset, the proposed method achieves a segmentation accuracy of 90.45% for the Car class, which demonstrates the method’s significantly superior segmentation performance. Additionally, the segmentation accuracy for the Building class reached 95.23%, surpassing the second-best method RS^3^Mamba by 0.38 percentage points. The improvement stems from the multi-scale, multi-directional convolutional kernel design in the strip feature extraction module, which precisely captures the strip features along building edges. Meanwhile, cross-directional attention enhances the global representation of boundaries and effectively resolves boundary fracture issues. On the TSRSD dataset, the F1 for water bodies reached 72.68%, significantly outperforming other comparison methods. Even if the indicators are slightly lower than the comparison algorithm in some categories, the proposed method demonstrates strong advantages in extracting strip objects, which effectively solves the problems of poor segmentation capability and discontinuous fracture, and exhibits robust performance even in complex backgrounds.

Nonetheless, our proposed method shows favorable performance in terms of parameter size in addition to achieving competitive segmentation accuracy. Unlike Swin Transformer [9] and CCTNet [43], which trade massive parameter stacking for performance, our proposed method achieves a superior mIoU with only 26.31 M parameters. In addition, our model shows some shortcomings in terms of FLOPs, GPU memory, and FPS.

#### 4.4.2. Qualitative Analysis

**Figure 5:**
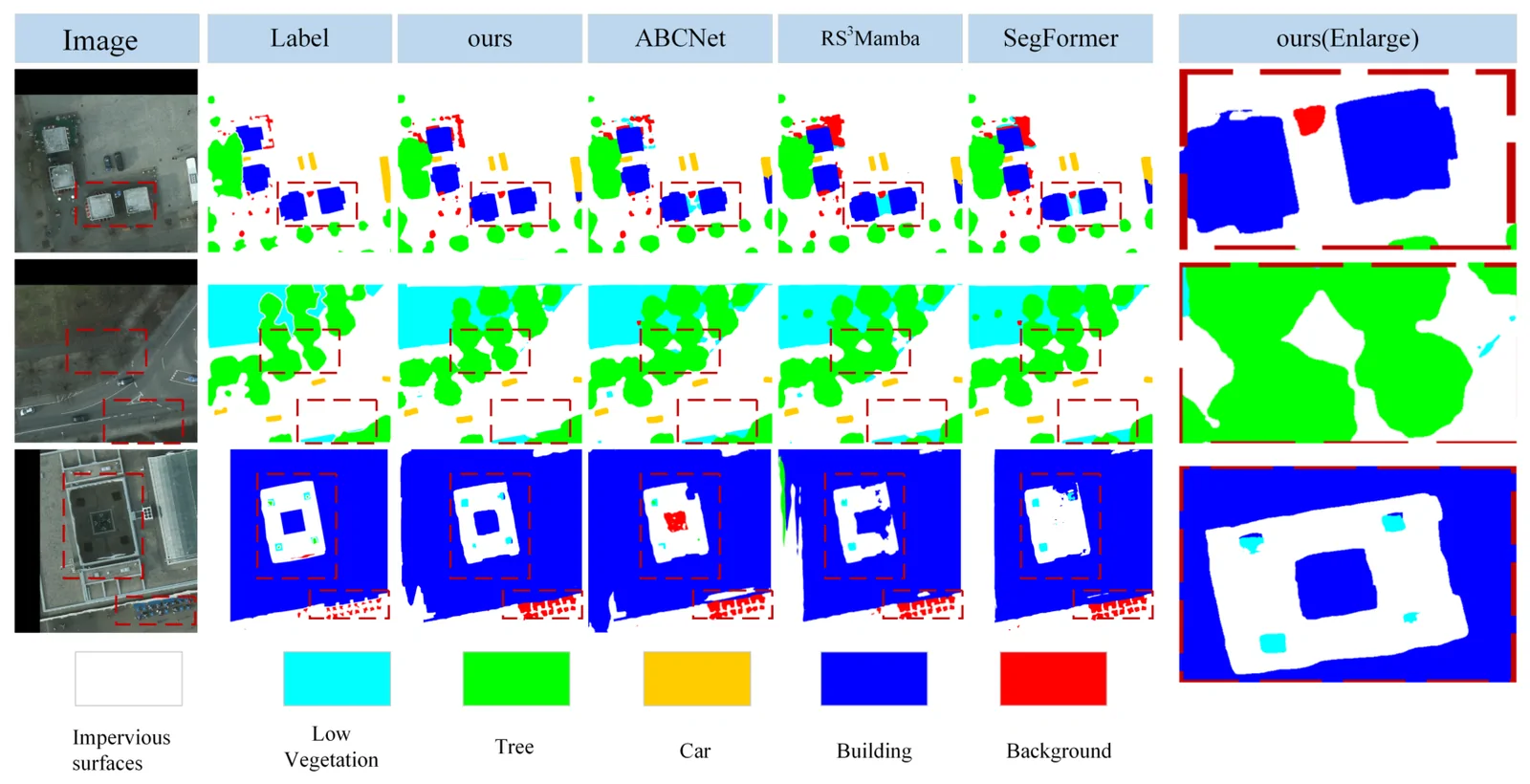
The segmentation results of different methods on the Potsdam dataset.

**Figure 6:**
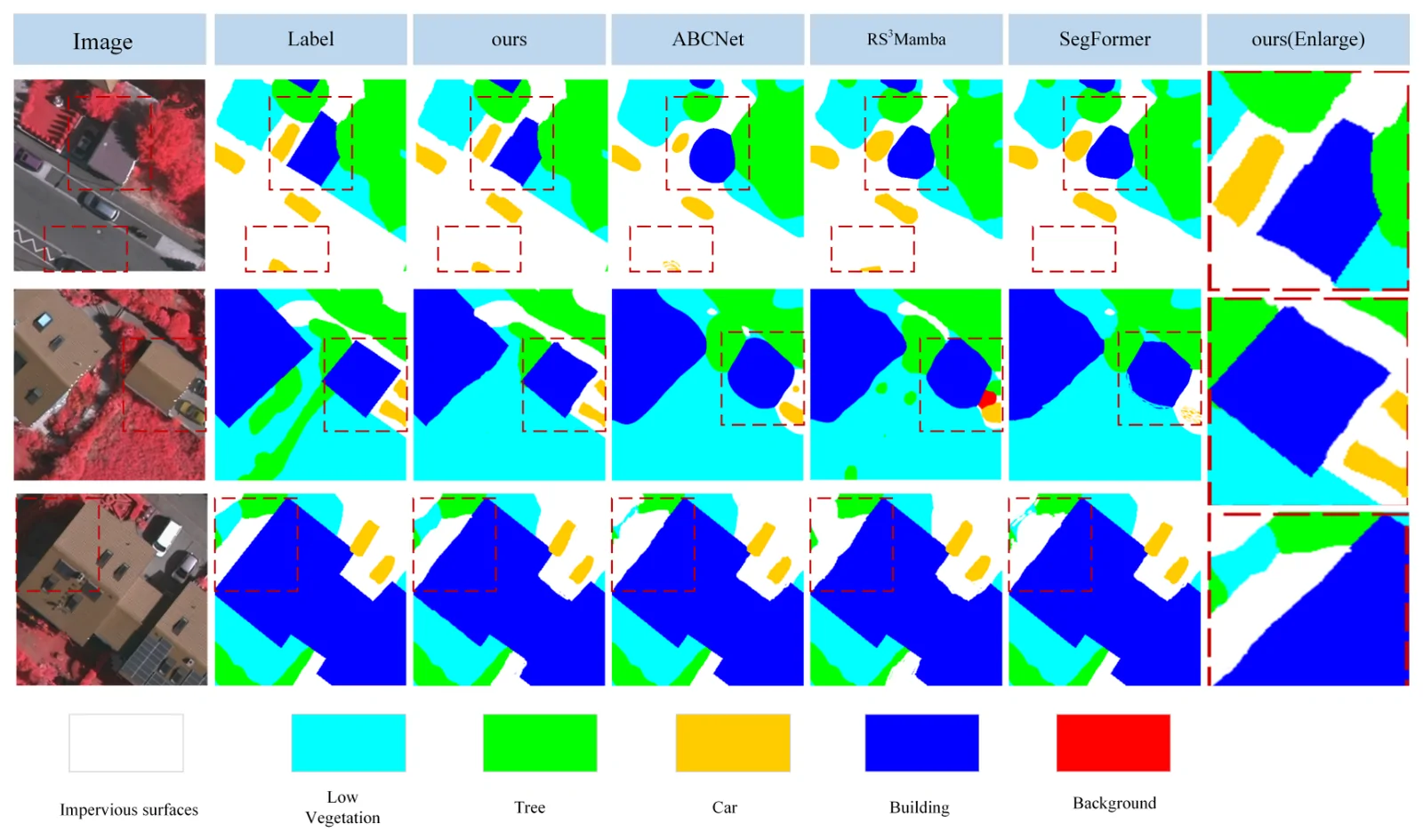
The segmentation results of different methods on the Vaihingen dataset.

**Figure 7:**
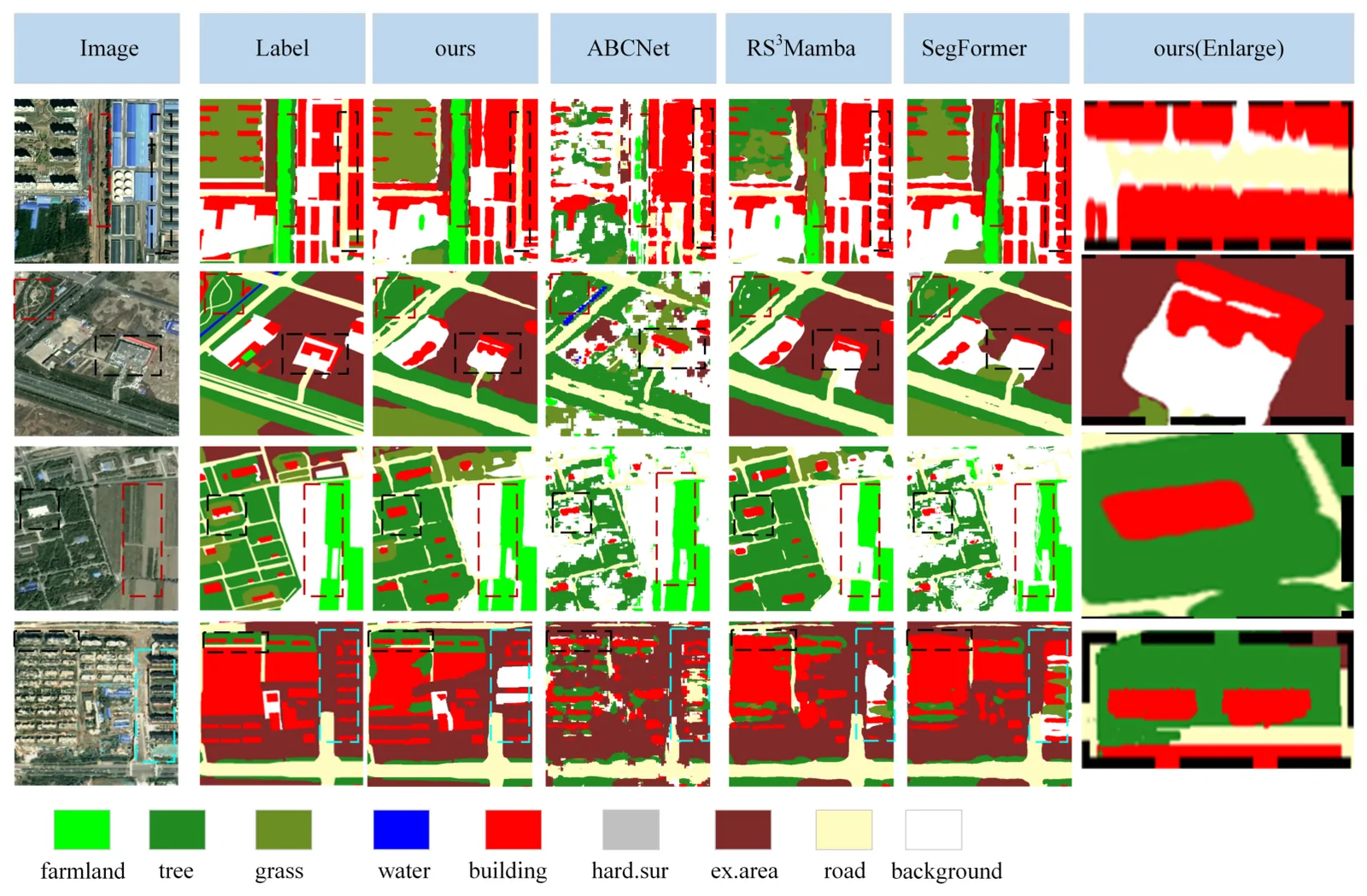
The segmentation results of different methods on the TSRSD dataset.

To visually validate the segmentation performance and advanced nature of the proposed method, we selected typical scene samples from the Potsdam, Vaihingen, and self-built TSRSD datasets. The segmentation results of the proposed method were visually compared with those of three representative comparison methods: ABCNet, RS^3^Mamba, and SegFormer, as shown in Figure 5, Figure 6 and Figure 7.

As observed in Figure 5, Figure 6 and Figure 7, our proposed method exhibits significant boundary continuity and fine-grained recognition in different scenarios. The proposed method demonstrates clear advantages in boundary delineation across multiple datasets. In the densely built-up areas of the Potsdam dataset, compared methods produce jagged edges and pixel confusion between buildings and impervious surfaces, distorting object contours. In contrast, our proposed method captures strip features along building edges through a dedicated strip feature extraction module, combined with auxiliary feature enhancement, which yields smooth and accurate boundaries that align well with ground truth. For the Vaihingen dataset, where boundaries are often blurred due to lighting and occlusion, baseline methods such as ABCNet show noticeable boundary softening and misclassification between low vegetation and trees. Our proposed method strengthens feature discrimination in boundary regions through superpixel-fused feature optimization. This results in clearly separated land-cover boundaries. On the TSRSD dataset, the proposed method accurately segments farmland, building, and road boundaries with high continuity, while the compared methods exhibit obvious fragmentation. Moreover, in vehicle-dense regions, SegFormer misses dispersed cars, and ABCNet generates incomplete outlines. In contrast, our proposed method integrates local features of scattered cars through adaptive fusion, which achieves full coverage of both clustered and isolated vehicles with accurately restored contours. The proposed method maintains the integrity of the form of small objects in the Car class. Overall, our proposed method produces notably sharper, more complete, and coherent boundaries, effectively validating the proposed method’s effectiveness in handling challenging boundary segmentation in remote sensing.

## 5. Conclusions

We propose a novel method centered on cross-directional aggregation and feature-loss optimization for remote sensing semantic segmentation. First, we model global contextual dependencies along both horizontal and vertical axes using dedicated directional convolution to capture strip structures better. Second, a dual-branch collaborative mechanism further strengthens long-range feature modeling. Finally, we fuse fine-grained superpixel details with global semantic information, systematically correcting edge errors and improving boundary continuity. Experimental results on the Potsdam, Vaihingen, and our self-built TSRSD datasets demonstrate that the proposed method achieves superior performance in object boundary integrity, small object accuracy, and segmentation continuity for strip objects, thereby confirming the effectiveness of our design. On the Potsdam, Vaihingen, and TSRSD datasets, our model achieves mIoU scores of 79.93%, 80.25%, and 64.89% and Recall scores of 87.70%, 88.94%, and 73.25%, respectively. Nevertheless, the method still has certain limitations: the superpixel preprocessing step introduces extra computational overhead under ultra-high-resolution input. For future work, we intend to explore end-to-end learnable superpixel generation to eliminate independent preprocessing, optimize network modules to further boost mIoU and Recall for shadow-affected regions, and adapt the model for real-world on-board remote sensing interpretation scenarios.

## Figures and Tables

**Table 1:** Ablation experimental results on the Potsdam dataset. Bold values indicate the best performance.

Method	Recall (%)	OA (%)	mIoU(%)
Base	77.32	80.64	74.81
Base + TCSFE	82.61	83.49	75.85
Base + HAGSI	81.97	83.02	75.87
Base + FDOSE	82.75	83.52	75.92
Base + TCSFE + HAGSI	83.12	84.54	77.81
Base + TCSFE + FDOSE	84.31	84.65	78.01
Base + HAGSI + FDOSE	85.51	85.56	78.12
Base + TCSFE + HAGSI + FDOSE	**87.64**	**86.77**	**79.92**

**Table 2:** Performance comparison of different feature extraction modules. Bold values indicate the best performance.

Feature Extraction Module	Recall (%)	OA (%)	mIoU (%)
-	74.12	75.54	68.81
FRH [33]	80.40	77.92	73.25
SFE [34]	73.85	76.35	72.91
DFEM [36]	78.03	80.52	75.60
CAFEM [35]	77.46	79.02	76.05
TCSFE (ours)	**83.09**	**81.61**	**79.87**

**Table 3:** Performance comparison of different auxiliary feature enhancement modules. Bold values indicate the best performance.

Auxiliary Feature Enhancement Module	Recall (%)	OA (%)	mIoU (%)
-	72.82	76.23	70.41
Swin [9]	80.33	80.99	73.60
DFM [37]	81.69	79.70	76.70
FIA [38]	83.10	82.13	71.36
HAGSI (ours)	**84.26**	**83.59**	**78.92**

**Table 4:** The impact of FDOSE on network performance. Bold values indicate the best performance.

Superpixel Edge Module	Recall (%)	OA (%)	mIoU (%)	UE	BR	ASA
-	83.12	84.54	77.81	0.2014	0.8321	0.9455
FDOSE (ours)	**87.64**	**86.77**	**79.92**	**0.1535**	**0.8736**	**0.9529**

**Table 5:** Performance comparison of different methods on the Potsdam dataset. The accuracy of the category is presented in the form of F1(%). Bold values indicate the best performance.

Methods	Imp.surf.	Car	Lowveg.	Tree	Building	mIoU (%)	mF1 (%)	Recall (%)	OA (%)	Params (M)	FLOPs (G)	Memory (G)	FPS
UNetFormer [33]	81.77	83.65	75.04	78.88	92.31	76.93	82.33	85.23	85.67	**11.68**	23.48	15.13	23
PSPNet [39]	80.54	83.22	76.18	81.31	91.77	75.80	82.60	75.71	75.06	24.17	45.12	18.05	25
Deeplabv3+ [40]	83.02	82.63	74.84	80.47	82.55	74.30	80.70	80.56	80.47	41.39	78.56	**13.45**	**32**
ABCNet [41]	80.91	87.01	76.86	79.59	92.39	75.95	83.35	82.26	88.74	14.15	**18.36**	15.42	26
RS^3^Mamba [42]	83.11	89.25	75.79	81.12	94.85	77.22	84.82	86.73	89.05	49.73	72.61	19.03	21
CCTNet [43]	81.23	83.12	75.89	79.62	93.11	72.39	82.59	84.03	88.12	35.27	35.79	15.32	20
Swin Transformer [9]	81.83	87.72	74.84	80.78	91.23	76.08	83.28	86.21	87.65	51.13	28.49	14.25	22
SegFormer [44]	83.92	86.18	76.21	81.03	91.87	76.44	83.84	86.90	89.38	26.53	32.24	17.24	18
LGBSwin [45]	83.15	85.53	76.59	80.92	90.35	78.67	83.31	85.13	88.64	66.53	55.23	19.57	19
SwinFAN [46]	84.17	87.69	**80.32**	**81.94**	90.87	79.45	84.99	86.79	86.97	97.20	57.95	18.36	31
Proposed method (Ours)	**84.34**	**90.45**	77.74	81.64	**95.23**	**79.93**	**85.88**	**87.70**	**89.76**	26.31	87.42	18.46	24

**Table 6:** Performance comparison of different methods on the Vaihingen Dataset. The accuracy of the category is presented in the form of F1(%). Bold values indicate the best performance.

Methods	Imp.surf	Car	Lowveg.	Tree	Building	mIoU (%)	mF1 (%)	Recall (%)	OA (%)	Params (M)	FLOPs (G)	Memory (G)	FPS
UNetFormer [33]	83.93	80.21	76.29	82.44	91.76	79.53	82.93	83.66	84.26	**11.68**	23.48	15.13	23
PSPNet [39]	81.36	83.10	73.74	83.95	93.52	69.53	83.13	76.53	85.40	24.17	45.12	18.05	25
Deeplabv3+ [40]	83.29	81.39	72.44	82.74	93.21	70.21	82.61	84.52	84.69	41.39	78.56	**13.45**	**32**
ABCNet [41]	80.72	80.75	72.07	83.01	92.56	73.62	81.82	88.81	84.41	14.15	**18.36**	15.42	26
RS^3^Mamba [42]	84.62	81.97	76.84	83.66	93.83	75.78	84.18	86.69	86.73	49.73	72.61	19.03	21
CCTNet [43]	82.12	80.92	73.08	83.14	92.93	72.64	82.44	84.18	82.57	35.27	35.79	15.32	20
Swin Transformer [9]	83.22	81.08	74.36	84.32	91.57	73.11	82.91	85.82	84.64	51.13	28.49	14.25	22
SegFormer [44]	83.21	80.63	76.53	84.45	93.19	74.40	83.60	86.53	86.25	26.53	32.24	17.24	18
LGBSwin [45]	82.32	81.47	75.79	81.48	92.75	75.67	82.76	85.73	85.94	66.53	55.23	19.57	19
SwinFAN [46]	**85.73**	81.83	**80.64**	82.73	91.37	77.51	84.46	87.64	86.83	97.20	57.95	18.36	31
Proposed method (Ours)	85.34	**83.13**	78.58	**83.94**	**94.27**	**80.25**	**85.05**	**88.94**	**87.32**	26.31	87.42	18.46	24

**Table 7:** Performance comparison of different methods on the TSRSD Dataset. The accuracy of the category is presented in the form of F1(%). Bold values indicate the best performance.

Methods	Farmland	Tree	Grass	Water	Building	Hard.sur	Ex.area	Road	mIoU (%)	mF1 (%)	Recall (%)	OA (%)	Params (M)	FLOPs (G)	Memory (G)	FPS
UNetFormer [33]	71.25	65.32	58.92	60.57	73.66	50.22	52.63	70.42	61.52	62.87	70.01	73.67	**11.68**	23.48	15.13	23
PSPNet [39]	69.52	67.81	56.04	61.35	69.24	52.47	51.04	68.35	60.65	61.98	66.33	69.76	24.17	45.12	18.05	25
Deeplabv3+ [40]	73.61	67.45	59.22	62.58	73.54	51.32	55.49	70.66	59.98	64.23	71.65	72.99	41.39	78.56	**13.45**	**32**
ABCNet [41]	68.47	64.75	53.21	64.66	70.38	53.64	51.27	68.84	60.26	61.90	70.58	71.36	14.15	**18.36**	15.42	26
RS^3^Mamba [42]	69.79	65.84	62.91	71.35	69.97	55.67	53.58	69.64	63.45	64.84	69.52	70.55	49.73	72.61	19.03	21
CCTNet [43]	70.56	66.42	59.86	69.61	66.81	59.37	55.64	68.94	62.53	64.65	70.51	68.74	35.27	35.79	15.32	20
Swin Transformer [9]	63.24	65.85	60.02	68.64	69.59	57.94	57.36	70.25	61.25	64.11	70.87	71.37	51.13	28.49	14.25	22
SegFormer [44]	66.74	69.56	58.65	66.37	69.21	58.64	56.89	70.01	62.69	64.50	69.85	69.97	26.53	32.24	17.24	18
LGBSwin [45]	67.24	66.35	59.26	67.53	69.75	57.81	57.93	70.32	61.37	64.52	69.61	70.85	66.53	55.23	19.57	19
SwinFAN [46]	73.69	69.01	61.23	70.00	72.54	**60.13**	**61.24**	70.87	63.57	67.34	72.53	74.62	97.20	57.95	18.36	31
Proposed method (Ours)	**74.96**	**70.65**	**64.33**	**72.68**	**74.85**	59.85	60.05	**71.64**	**64.89**	**68.63**	**73.25**	**75.69**	26.31	87.42	18.46	24

## Data Availability

We used two public 2D semantic labeling datasets, Vaihingen and Potsdam, provided by the International Society for Photogrammetry and Remote Sensing (ISPRS), https://www.isprs.org/resources/datasets/benchmarks/UrbanSemLab (accessed on 25 September 2026). The self-built Taiyuan Satellite Remote Sensing Dataset (TSRSD) presented in our study will be made publicly available on GitHub upon acceptance of the manuscript.
